# Predicting chronic responses to calcium channel blockade with a virtual population of African Americans with hypertensive chronic kidney disease

**DOI:** 10.3389/fsysb.2024.1327357

**Published:** 2024-07-04

**Authors:** John S. Clemmer, W. Andrew Pruett, Robert L. Hester

**Affiliations:** ^1^ Department of Physiology and Biophysics, University of Mississippi Medical Center, Jackson, MS, United States; ^2^ HC Simulation, LLC, Canton, MS, United States

**Keywords:** African American, hypertension, kidney disease, physiological model, GFR, glomerular filtration rate

## Abstract

Chronic kidney disease (CKD) is associated with the progressive loss of functional nephrons and hypertension (HTN). Clinical studies demonstrate calcium channel blocker (CCB) therapy mitigates the decline in renal function in humans with essential HTN. However, there are few long-term clinical studies that determine the impact of CCBs in patients with hypertensive CKD. African Americans (AA) have a higher prevalence of CKD and a faster progression to total kidney failure as compared to the white population but the mechanisms are poorly understood. Both clinical evidence (the African American Study of Kidney Disease and Hypertension, or AASK trial) and experimental studies have demonstrated that CCB may expose glomerular capillaries to high systemic pressures and exacerbate CKD progression. Therefore, using a large physiological model, we set out to replicate the AASK trial findings, predict renal hemodynamic responses and the role of the renin-angiotensin system during CCB antihypertensive therapy in a virtual population, and hypothesize mechanisms underlying those findings. Our current mathematical model, HumMod, is comprised of integrated systems that play an integral role in long-term blood pressure (BP) control such as neural, endocrine, circulatory, and renal systems. Parameters (n = 341) that control these systems were randomly varied and resulted in 1,400 unique models that we define as a virtual population. We calibrated these models to individual patient level data from the AASK trial: BP and glomerular filtration rate (GFR) before and after 3 years of amlodipine (10 mg/day). After calibration, the new virtual population (n = 165) was associated with statistically similar BP and GFR before and after CCB. Baseline factors such as elevated single nephron GFR and low tubuloglomerular feedback were correlated with greater declines in renal function and increased glomerulosclerosis after 3 years of CCB. Blocking the renin-angiotensin system (RAS) in the virtual population decreased glomerular pressure, limited glomerular damage, and further decreased BP (−14 ± 8 mmHg) as compared to CCB alone (−11 ± 9 mmHg). Our simulations echo the potential risk of CCB monotherapy in AA CKD patients and support blockade of the renin angiotensin system as a valuable tool in renal disease treatment when combined with CCB therapy.

## Introduction

Renal function is the primary determent of long-term blood pressure (BP) control; and hypertension (HTN) is the most frequent comorbidity of chronic kidney disease (CKD), affecting up to 80% of these patients (Whaley-Connell et al., 2008). African Americans (AA) have more HTN, are less likely to have controlled BP, and 4x more likely to progress to complete kidney failure as compared to the white population (Hardy et al., 2021), even after controlling for social determinants of health (Abrahamowicz et al., 2023). Observational studies demonstrate uncontrolled BP to be a strong independent risk factor for CKD progression. However, the African American Study of Kidney Disease and Hypertension (AASK) trial suggests AA with CKD may have detrimental responses to calcium channel blockers (CCB) regardless of BP.

While the benefits of CCB treatment on BP control and chronic renal function are well established in essential HTN (Bidani et al., 1987), animal models suggest that low renal mass can exacerbate the transmission of systemic pressures and damage glomerular capillaries (Bidani et al., 1987). Indeed, many investigators have shown that preglomerular vasodilation with CCB can accelerate glomerulosclerosis and CKD progression (Bidani et al., 1987; Neuringer and Brenner, 1993; Griffin et al., 1994; Griffin et al., 1995; Griffin et al., 1999). Despite these data, CCBs are one of several first-line antihypertensive therapies currently recommended for hypertensive CKD treatment (Pugh et al., 2019), making the current strategies for at risk populations, such as AA with hypertensive CKD unclear.

Reducing intraglomerular pressure plays a critical role in decreasing the chronic decline of renal function. There are well-known renal benefits in CKD when using renin-angiotensin system (RAS) blockade. RAS blockade vasodilates the efferent arterioles, lowers glomerular pressure, and slows CKD progression, even when given with a dihydropyridine CCB (Bakris et al., 2010; Kobori et al., 2013; Richardson et al., 2021). However, CCB and RAS blockade combination therapy is largely untested in AA CKD patients. Additionally, tubuloglomerular feedback (TGF) is a major renal mechanism that plays a critical role in maintaining constant glomerular pressures despite fluctuating systemic pressures and may become increasingly impaired as functional nephron numbers become compromised (Bidani et al., 1987). However, there have been no experimental or clinical studies that directly measure glomerular hemodynamics, TGF, single nephron GFR, glomerular pressure, or glomerulosclerosis during chronic CCB therapies in CKD.

Mathematical modeling provides the ability to analyze complicated interrelated effects across multiple physiological systems that are difficult to test experimentally. Our current model, HumMod, is developed by the University of Mississippi Medical Center and has been previously used to model the effects of CCB-induced glomerular HTN in a single model of CKD (Moore and Clemmer, 2021). Using our laboratory’s technique to create virtual populations (Clemmer et al., 2021), we generated an *in silico* trial of CCB in AA with hypertensive CKD. The major goal of this study was to validate a population of models that are statistically similar to the CCB arm of the AASK trial and predict population responses to adjunctive RAS inhibition. We hypothesized that in a population model of hypertensive CKD, CCB would increase nephron damage by exacerbating glomerular pressures.

## Methods

### Clinical data

The current study used data from the AASK trial, a US multicenter, randomized control trial of 1094 AA aged 18–70 years old with HTN and CKD with measured glomerular filtration rate (GFR) 20–65 mL/min (Wright et al., 2002; Appel et al., 2010). Follow-up was 3–6 years and included a treatment arm using a dihydropyridine CCB, amlodipine (5–10 mg/day). Individual patient-level data were obtained through NIDDK Central Biorepository. Only patients with baseline and 3-year data for seated BP and GFR were included (*n* = 165). GFR was assessed by iothalamate clearance, and BP was measured using a random zero sphygmomanometer three consecutive times on each visit after patient was quietly seated for at least 5 min. The BP value was an average of the last 2 BP measurements. Data from two initial visits were averaged for each patient’s baseline values while measurements from months 30 through 42 were averaged and used for the 3-year datapoints for the virtual population.

#### Physiological model

All simulations were performed with HumMod, an integrated model of human physiology composed of mathematical relationships based upon well-understood cell and tissue physiology and derived from experimental and clinical data. This model accurately reproduces many pathophysiological states (Pruett et al., 2016; Clemmer et al., 2017; Clemmer et al., 2019; Clemmer and Pruett, 2022) and has been used previously to examine mechanisms of CKD progression in a single model (Moore and Clemmer, 2021). This study builds upon this previous work and creates a virtual population with 165 different parameterizations of HumMod to replicate and understand physiological variability and predict cardiovascular and renal hemodynamic variables that are difficult or impossible to measure in a human study. The details of the model structure are beyond the scope of this article, and have been previously described. The model and its code are freely available for academic download as a single ZIP file at http://hummod.org/ccb-population.zip. Additionally, Supplementary Material including instructions on how to run the experimental protocols can be found at: https://doi.org/10.6084/m9.figshare.24325067.v3.

Briefly, HumMod contains organ systems that make up the peripheral circulation and consists of two kidneys, left and right heart, skeletal and respiratory muscle, gastrointestinal tract, liver, bone, brain, fat, skin, lungs, and a miscellaneous tissue blood flow (∼400 mL/min) that represents the sum of the remaining smaller organs. Organ blood flow is controlled by the pressure gradients and the vascular resistance of the organ’s circulation that’s independently regulated by local tissue factors such as oxygen partial pressure, the sympathetic nervous system (SNS), angiotensin II (Ang II), and treatments with direct effect on the vasculature such as amlodipine (Supplementary Figure S12).

Cardiac function is modeled after the Suga and Sagawa mathematical heart model (Suga and Sagawa, 1972). Total ventricle mass is divided into right and left ventricles (LV). Hypertrophy of the LV is determined by diastolic and systolic stresses. LV stress is calculated under the assumption of an ellipsoidal LV geometry as previously described (Grossman et al., 1975).

The kidneys are separated into vascular and nephron components. The renal vasculature in the model includes renal artery, interlobar artery, and afferent and efferent arterioles. Factors that influence both afferent and efferent resistances and tubular sodium reabsorption within each tubular segment (i.e., proximal tubule, loop of Henle, distal tubule, and collecting duct) include physical factors, SNS, hormones, and TGF. TGF is increased in the model by increased sodium delivery at the macula densa and increased plasma Ang II (Supplementary Figure S11). TGF has a direct vasoconstrictive effect at the afferent arteriole based on rodent experimental studies (Casellas and Moore, 1990; Schnermann et al., 1990). Specific descriptions of these relationship are shown in the Supplementary Material and in previous publications (Clemmer et al., 2017; Clemmer et al., 2018).

Glomerulosclerosis was modeled as damaged (non-filtering) nephrons determined by a delayed reduction of nephron number as a function of glomerular capillary pressure (P_c_):

$$\#Nonfiltering\;Nephrons=\int_{0}^{T}f\left(P_{C}\right)dt$$

Nephron damage is initiated at 70 mmHg glomerular pressure and increases at higher pressure and is implemented with a time delay of 120 days (Supplementary Figure S1). This function was derived from the 10%–20% increase in baseline glomerular pressure needed for glomerular damage in animals (Tapia et al., 2012; Fan et al., 2021), and the delay constant was chosen to match the overall renal function decline observed in the CCB arm of the AASK trial (i.e., −1 to −4 mL/min/year).

Gut permeability and clearance rate of amlodipine in the model were fit to match pharmacodynamics reported in a mostly black population administered 10 mg of amlodipine (Rohatagi et al., 2008) (Figure 3). The peripheral and renal vasodilatory effects of amlodipine are based on chronic amlodipine therapy in humans with CKD (Konoshita et al., 2010) as well as amlodipine treatment in essential HTN patients (Delles et al., 2003; Lindqvist et al., 2007) and shown in (Supplementary Figures S4 and S12).

#### Virtual population

**FIGURE 1:**
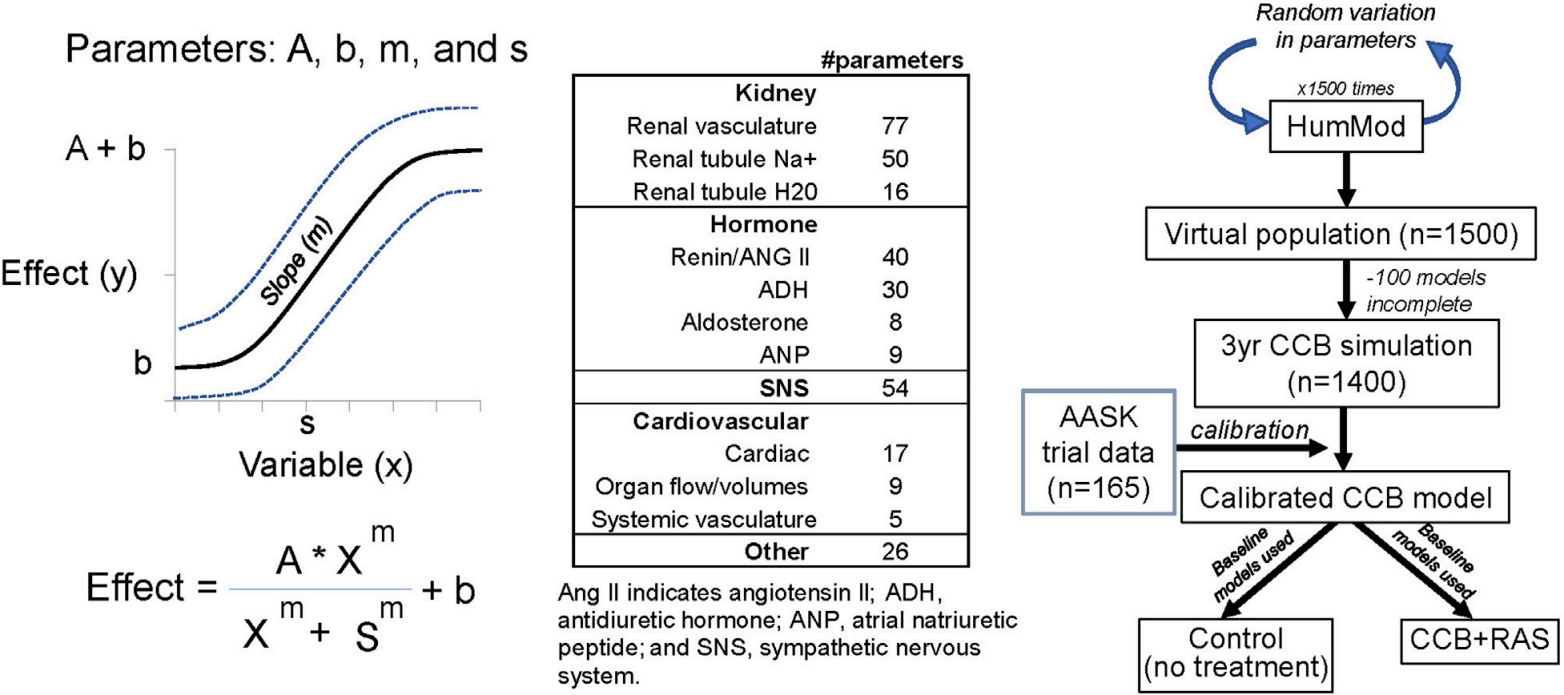
Parameters used to create sigmoid relationships within model, breakdown of all varied parameters, and protocol for creating virtual population.

**FIGURE 2:**
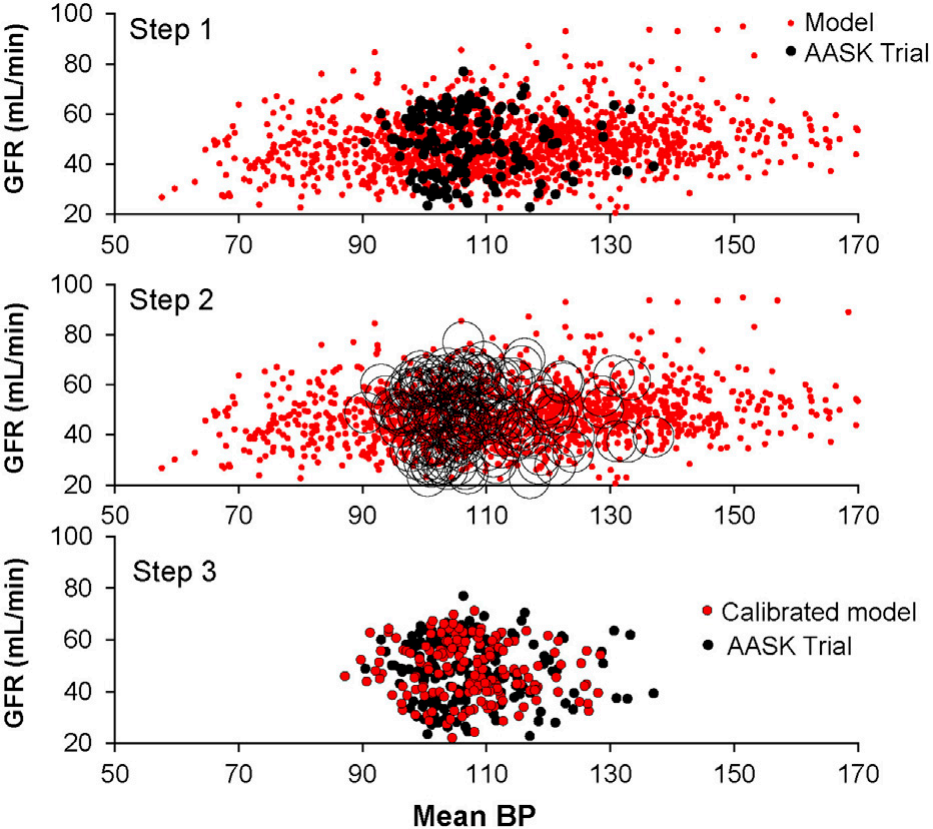
Calibration of virtual patients (*n* = 1,400) using clinical data (*n* = 165): glomerular filtration rate and mean arterial pressure before (shown) and after calcium channel blockade (not shown).

All cardiovascular and renal parameters (*n* = 341) in HumMod were varied 5% (Figure 1). In addition, parameters representing baseline renin and aldosterone secretion, sympathetic nerve activity, heart rate, baroreceptor sensitivity, salt intake, amlodipine clearance, sensitivity of the myogenic mechanism, renal vascular resistance, as well as baseline proximal and distal tubule sodium reabsorptions were varied up to 100%. Most parameters represented simple multiplicative constants such as salt intake or hormone secretion constants. Parameters in the model also describe sigmoid relationships (*A, b, m,* and *s*) as shown in Figure 1. The complete list of parameters varied and their ranges within the model can be found in the Supplementary Material. These parameter sets were randomly varied 1,500 times, representing the initial uncalibrated population. Once loaded into the model, the experimental protocol was executed. Models were then selected with k-nearest neighbors which is a non-parametric supervised machine learning algorithm (Figure 2). Each model datapoint within 1 standard deviation of a clinical datapoint (in each of the four variable dimensions: BP and GFR before and after CCB) were sampled from the uncalibrated population. Each selected model (parameter set) was again varied 10 times (up to 5%) to yield a new population of models that were then subject to the same experimental protocol. This process decreases the likelihood that a model is near the clinical data just by chance and increases the robustness of the population. This process was repeated until the selected virtual population was statistically similar to the clinical data. All parameter set variations and model calibration functions were performed using Mathematica (Version 12, Champaign, IL).

### Simulation protocols

Sodium intake was set to normal levels of 123 ± 53 mEq/day corresponding to the salt intake range reported in the AASK Trial (90–258 meq/day) (Pearson et al., 2020). Additionally, the effects of low salt diet were assessed by lowering salt intake to 90 mEq/day (Supplementary Figure S2). Water intake was determined by the thirst mechanism in the model (i.e., serum osmolarity).

To reach a baseline CKD similar to the AASK trial population, parameter sets were loaded and baseline nephron counts were slowly reduced 70% ± 10% over the course of 9 months to ensure steady-state conditions. For 3 years, 10 mg of amlodipine was given orally once per day. Responses to no treatment (Control), CCB, or CCB and RAS blockade were simulated during CKD with different perturbations:

1. Control: Baseline model was simulated for 3 years without treatment
2. CCB: 10 mg amlodipine per day for 3 years
3. CCB + RAS: 10 mg amlodipine per day for 3 years with 80% RAS blockade

Explicit details on how to execute these experimental protocols in the model are provided in the model download or Supplementary Material.

### Statistical analysis

Data were summarized using mean ± standard deviations unless otherwise noted. Data was analyzed with unpaired nonparametric *t*-test (Mann-Whitney two-sided) for comparing virtual and clinical data. When comparing between virtual trials (control, CCB, and CCB + ACE) a paired *t*-test (Wilcoxon matched pairs signed rank test) was used when comparing the same 165 models between groups or two-way ANOVA repeated measures when including the effects of time (Figure 6). Similarly, delta changes were also compared between groups using the paired Wilcoxon test (Figures 5, 6). The nonparametric Spearman test was used to detect significant correlations as shown in Figure 5 and Figure 7. All statistical analyses were performed using GraphPad Prism 10 (La Jolla, CA). Probability was based on two-tailed tests of significance, and significance was considered *p* < 0.05.

## Results

**FIGURE 3:**
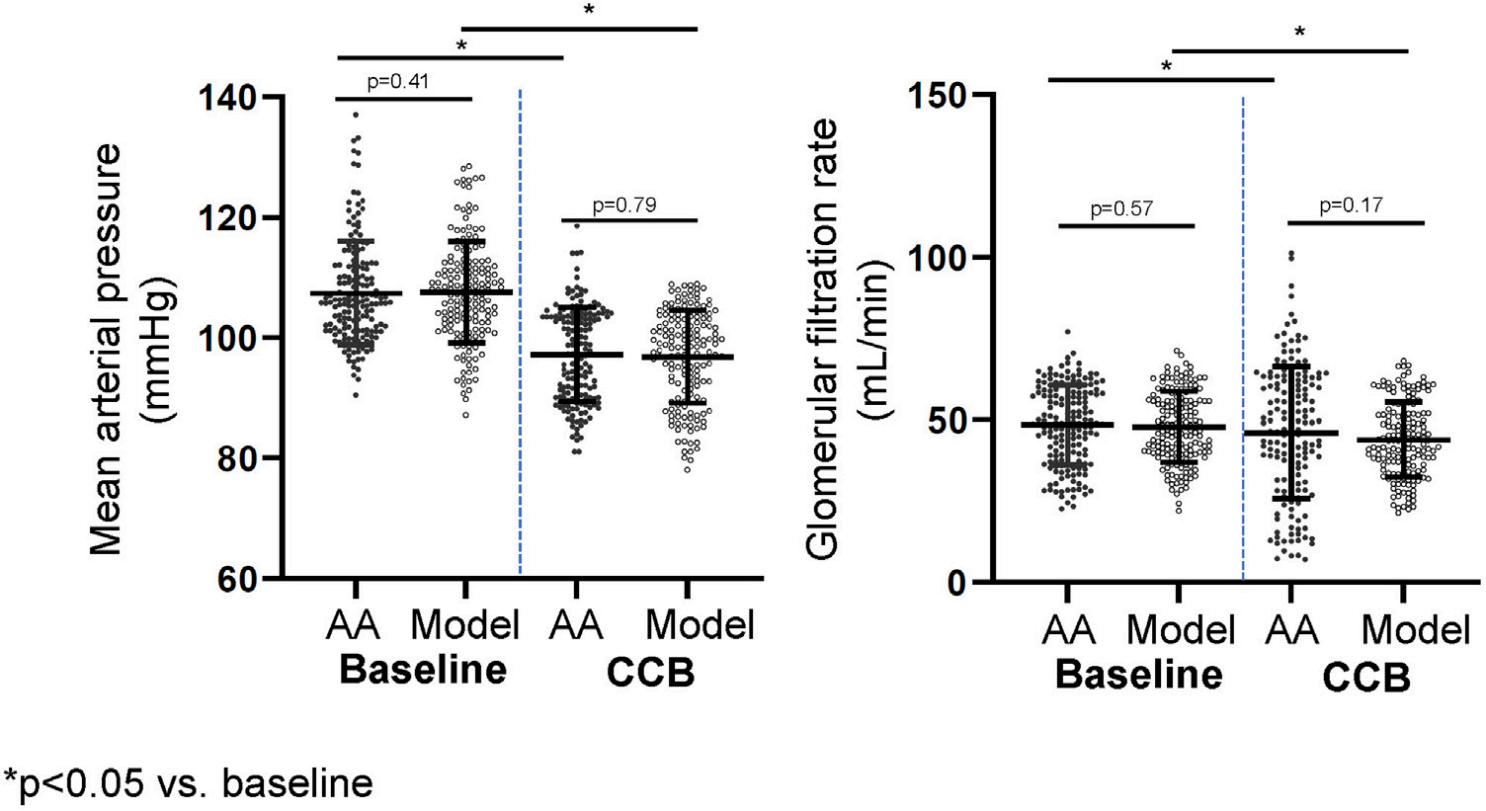
Mean arterial pressure and glomerular filtration rate before and after calcium channel blockade (3 years).

In the calibrated virtual population, baseline CKD was associated with a baseline nephron count 720,268 ± 182,818 (a reduction of 70% below the normal model value of 2,400,000) (Table 1). This was accompanied by HTN (130 ± 11 mmHg and 94 ± 10 mmHg systolic and diastolic BP, respectively). Due to the low nephron mass, the CKD model was also associated with increased renal vascular resistance, single nephron GFR (snGFR), and glomerular pressure but relatively similar angiotensin II and extracellular fluid (ECF) volume (Table 1) as compared to normal conditions. The baseline mean BP (108 ± 8 mmHg) and GFR (48 ± 11 mL/min) in the virtual population was statistically similar to the AA population seen in the AASK Trial (Figure 3; Table 1). Interestingly, although naive to the calibration process, LV mass was not statistically different as compared to the AASK trial (253 ± 22 vs. 246 ± 77, Table 1).

**TABLE 1:** Baseline cardiovascular and renal variables in the virtual population and responses to CCB treatment and comparisons to clinical data (AASK Trial).

		Baseline CKD		CKD + CCB		Change with CCB		Change with CCB + RASi
	Normal	Model	AASK trial	Model	AASK trial	ΔModel	AASK trial	ΔModel
MAP (mmHg)	94	108 ± 8	107 ± 9	97 ± 7	97 ± 8	−11 ± 9	−10 ± 11	−14 ± 8#
GFR (mL/min)	129	48 ± 11	48 ± 12	44 ± 11	46 ± 20	−3.9 ± 9	−2.6 ± 15	−4.3 ± 7
RBF (mL/min)	1,100	382 ± 113		368 ± 116		−14 ± 70		−2 ± 56#
Aff Art R (mmHg/mL/min)	0.07	0.25 ± 0.11		0.19 ± 0.11		−19% ± 40%		−31% ± 23%#
Eff Art R (mmHg/mL/min)	0.11	0.43 ± 0.20		0.50 ± 0.45		12% ± 42%		1% ± 21%#
SNGFR (nL/min)	54	69 ± 16		72 ± 14		7% ± 14%		1% ± 15%#
Glomerular pressure (mmHg)	52	68 ± 8		69 ± 7		2% ± 6%		−1 ± 7%#
TGF (x Normal)	1	0.76 ± 0.3		0.66 ± 0.4		−10% ± 16%		−29% ± 18%#
Nephron # (million)	2.4	0.72 ± 0.2		0.64 ± 0.2		−11% ± 9%		−8 ± 6%#
Chronic GFR decline (mL/min/yr)						−1.1 ± 2	−2.4 ± 5	−0.5 ± 2#
ANG II (pg/mL)	12	14 ± 21		12 ± 18		−2 ± 7		−7 ± 11#
Aldosterone (ng/dL)	11	21 ± 9		20 ± 9		−1 ± 4		−3 ± 4#
ANP (pmol/L)	27	21 ± 21		36 ± 41		15 ± 27		3 ± 12#
ECFV (L)	15	15 ± 0.6		15 ± 0.6		0.3% ± 2%		−1 ± 2%#
LV mass (g)	223	253 ± 22	246 ± 77	243 ± 15	222 ± 74*	−3.4% ± 5%	−3.7% ± 24%	−5.9% ± 6%#

CKD, indicates chronic kidney disease; MAP, mean arterial pressure; GFR, glomerular filtration rate; RBF, renal blood flow; Aff Art R, afferent arteriolar resistance, Eff Art R, efferent arteriolar resistance; SNGFR, single nephron GFR; TGF, tubuloglomerular feedback; ANG II, angiotensin II; ANP, atrial natriuretic peptide; ECFV, extracellular fluid volume; and LV, right ventricle. Normal model represents a single model with normal baseline values. All variables were statistically different between baseline CKD, and CKD + CKD, models. Change represents absolute value unless denoted with %. Change in CCB, and CCB + ACE, responses represent changes at 1 year. Chronic GFR, decline represents decline from 6 to 36 months *p < 0.05 vs. Model; #*p* < 0.05 vs. CCB, change.

Acute changes in response to 10 mg amlodipine in the virtual population are shown in Figure 3. ECF concentrations of amlodipine reached steady-state in approximately 1 week with a trough (minimum) concentration of 9.5 ± 3 ng/mL, similar to Rohatiagi et al. (Rohatagi et al., 2008) (Figure 3). Mean systemic BP significantly decreased during the first week of amlodipine dosing, and there were acute increases in GFR and glomerular pressure after each dose, initially. However, overall glomerular pressure fell as the systemic BP decreased during the first week of CCB therapy (Figure 3). Individual model responses for the first week of CCB therapy are shown in Supplementary Figure S3.

**FIGURE 4:**
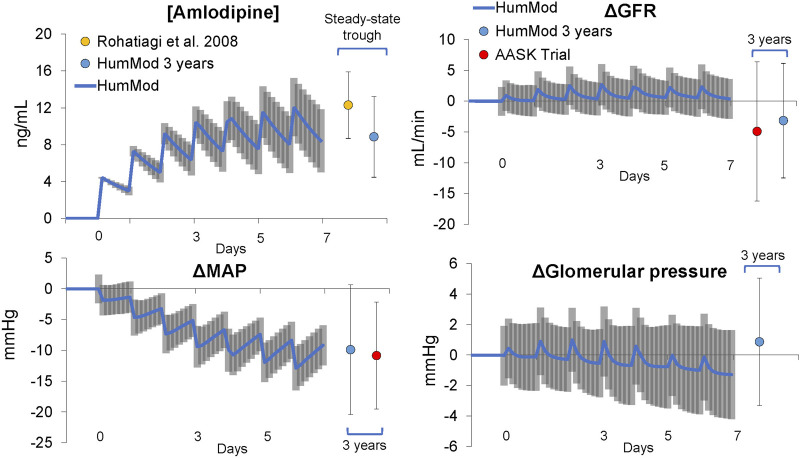
Acute and chronic responses to calcium channel blockade (amlodipine, 10 mg/day) in virtual and clinical patients.

**FIGURE 5:**
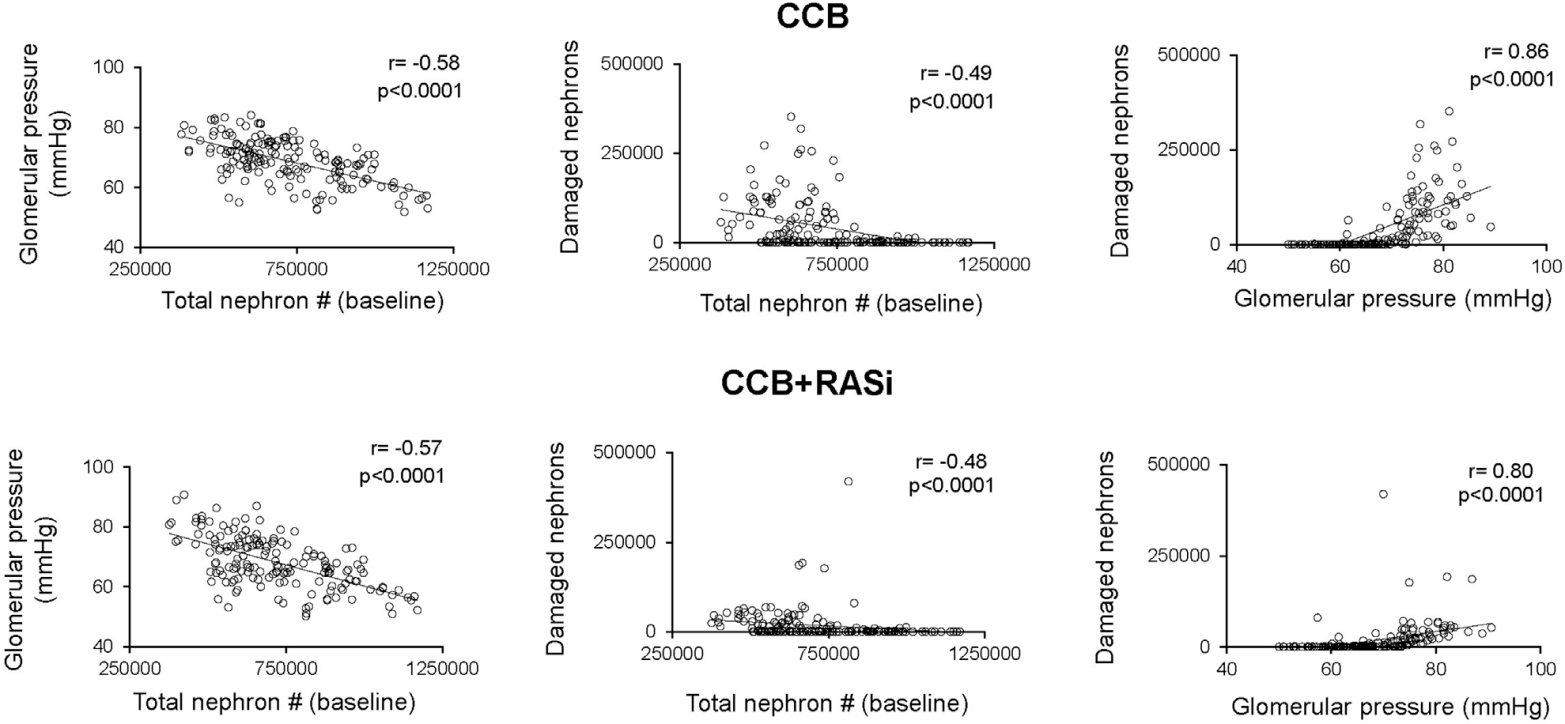
Relationship between baseline nephron number, baseline glomerular pressure, and damaged nephrons in virtual patients treated 3 years with calcium channel blocker with or without renin angiotensin system inhibition.

**FIGURE 6:**
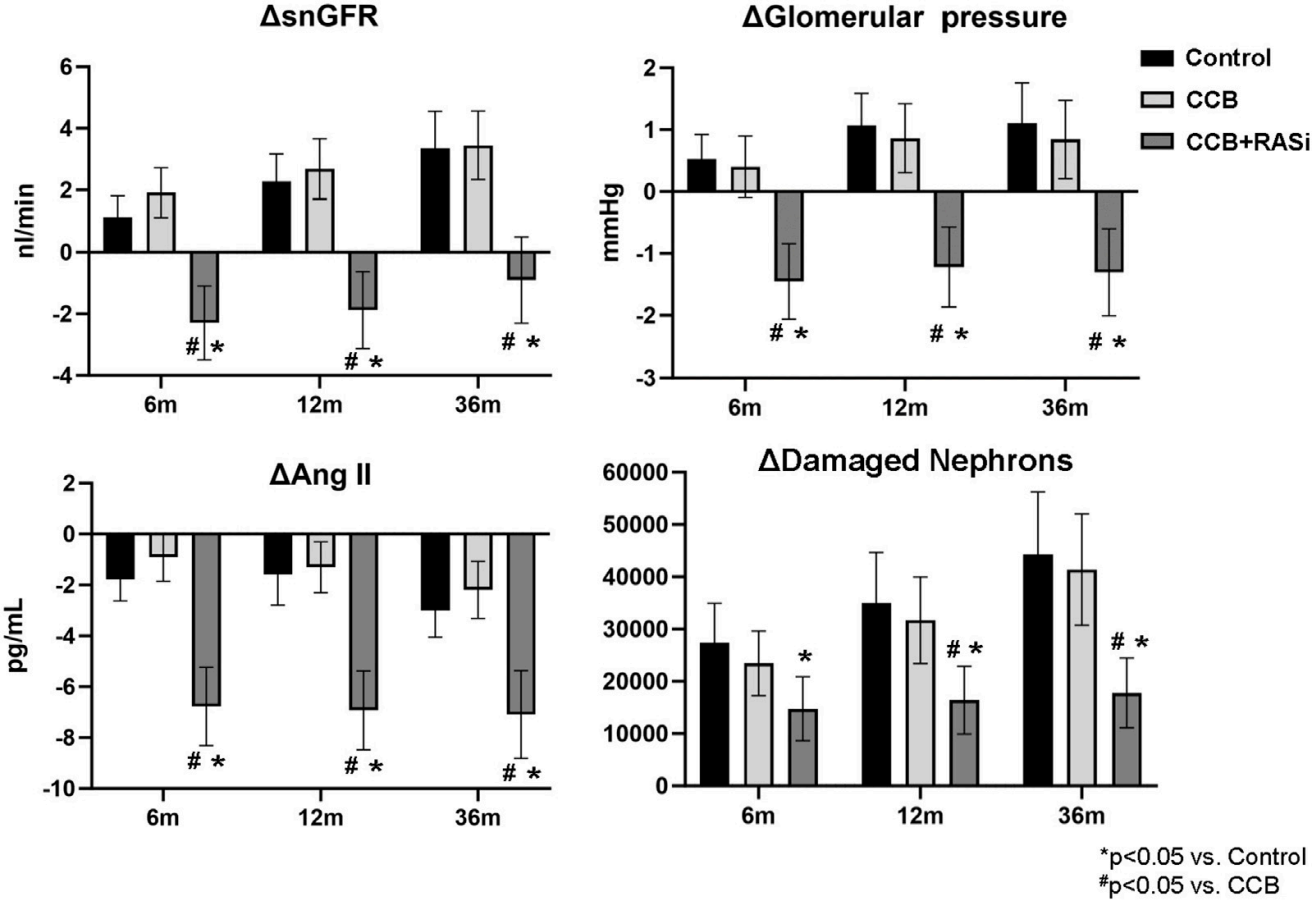
Changes in glomerular hemodynamics, angiotensin II, and damaged nephrons with and without renin angiotensin system inhibition with mean ± 95% CI shown.

**FIGURE 7:**
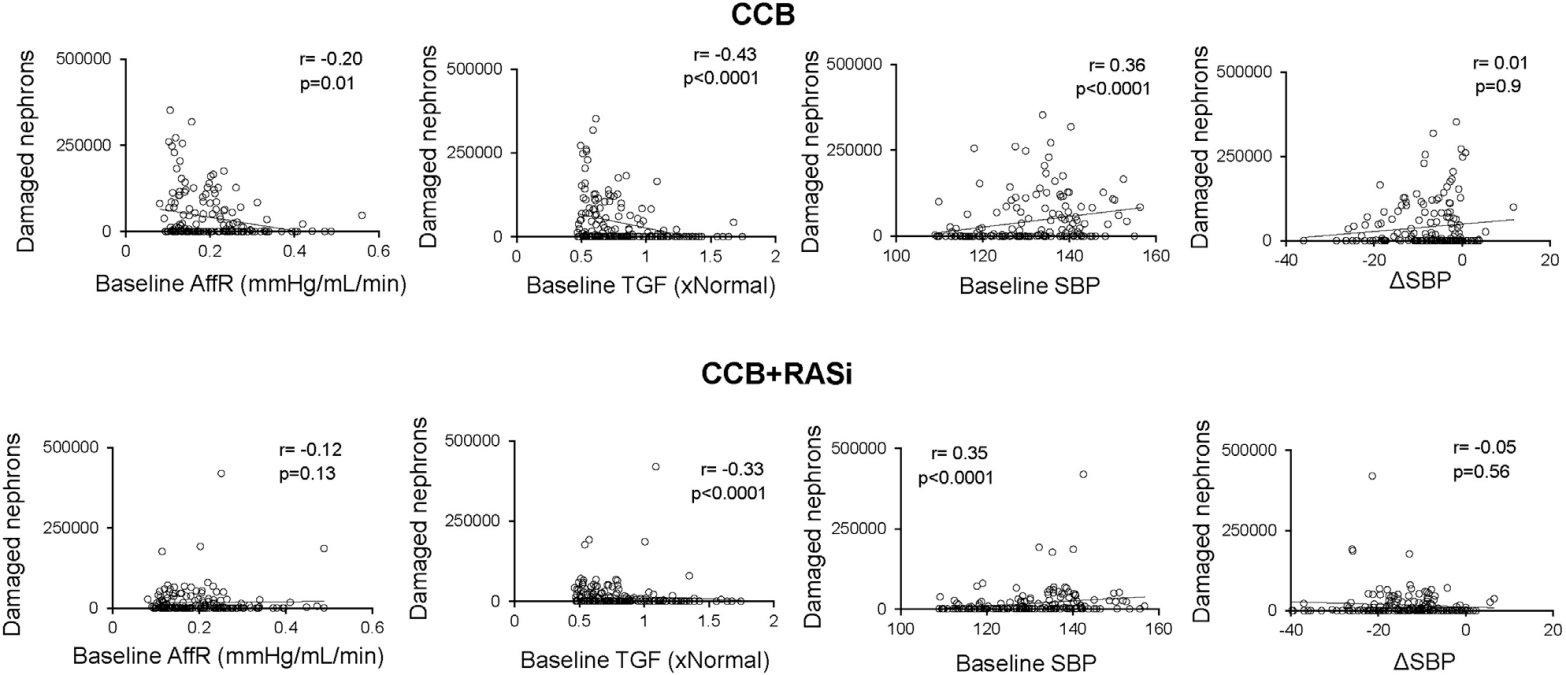
Relationships between baseline nephron number, glomerular pressure, and glomerulosclerosis in virtual patients treated with calcium channel blocker with or without renin angiotensin system inhibition. Damaged nephron count represents damaged nephron count at 3 years.

Chronic CCB therapy was associated with statistically significant decreases in mean BP and GFR in both the virtual population and AASK trial participants (Table 1). Both the mean BP and GFR in the virtual and real population were statistically similar to each other after 3 years of therapy (Figure 4). The fall in systemic BP after CCB was associated with significantly lower afferent arteriolar resistance and LV mass in the virtual population (Table 1). There were less nephrons after 3 years of CCB therapy corresponding to 41 ± 69 thousand damaged nephrons. This was significantly correlated with significantly increased glomerular pressure (2% ± 6%) and snGFR (7% ± 14%) after CCB (Figure 5). Moreover, low *baseline* nephron number was negatively correlated with both glomerular pressure and damaged nephrons after CCB (Figure 5). As compared to baseline, Ang II was decreased after 3 years of CCB therapy due to the separate influences of increased sodium delivery to the macula densa, increased in plasma ANP, and decreased functional nephron number (Table 1). In the CCB simulation, there were no significant differences in the change in snGFR, glomerular pressure, or Ang II as compared to the Control simulation (3 years without any treatment) (Figure 6). The increase in damaged nephrons with CCB treatment was correlated with baseline afferent arteriolar resistance, baseline TGF, and baseline BP (Figure 7). TGF was decreased at baseline and further reduced with CCB therapy (Table 1). Interestingly, the damaged nephron count was lower in the virtual population with the greatest BP-lowering effects from CCB. With the addition to RAS inhibition to the CCB model, there were significantly greater falls in mean BP and afferent arteriolar resistance but lower efferent arteriolar resistance, snGFR, glomerular pressure, Ang II, and nephron damage as compared to the CCB simulations (Table 1; Figure 6). TGF after RAS inhibition was significantly reduced (−29%) (Table 1).

## Discussion

There are few controlled non-diabetic clinical trials examining the role of CCB in hypertensive CKD while also considering the impact of race. Also, clinical evidence and recommendations for first-line therapy in nondiabetic CKD patients (without proteinuria) is unclear. The current study uses a mathematical model to predict chronic responses to CCB during low renal function in a virtual AA population. Here we present a novel approach that highlights important factors during antihypertensive treatment in CKD independent from decreases in systemic BP. To our knowledge, this is the first ever *in silico* trial simulated by a whole-body physiological model trained with patient-level data. These virtual trials predict the temporal changes in renal function during CCB with or without RAS blockade and demonstrates the important role for efferent arteriolar conductance in slowing CKD progression. Interestingly, although baseline TGF was associated with greater nephron damage, RAS blockade ameliorated the glomerular HTN and preserved functioning nephrons despite impairing TGF further. Moreover, we show that the decrease in overall GFR may not reflect the impact on functional nephrons and renoprotection. Indeed, CCB + RAS blockade resulted in lower GFR as compared to CCB alone, but this occurred while glomerular hyperfiltration and nephron damage were significantly blunted.

TGF is a main autoregulatory mechanism in the kidney to buffer changes in renal perfusion pressure, i.e., an increase in TGF occurs in response to increased renal perfusion pressure or sodium filtration and is evidenced by a subsequent vasoconstriction in preglomerular vasculature. In the current study, models with impaired (decreased) baseline TGF (and consequently a decreased preglomerular resistance) were significantly correlated with greater nephron damage in both CCB and CCB + RAS simulations (Figure 7). Further, TGF was actually reduced with CCB (−10% in the TGF effect on afferent arteriolar tone) and further impaired with CCB + RAS blockade simulation (Table 1). These simulations suggest that baseline TGF is impaired in these mathematical models of CKD, and the amelioration of nephron damage with CCB + RAS blockade was not through modulation of TGF. Indeed, in early animal studies, Ang II was shown to increase TGF magnitude and sensitivity whereas RAS blockade attenuates TGF responsiveness (Mitchell and Navar, 1988; Braam et al., 1995). In a recent animal study, low nephron mass was associated with impaired TGF and increased glomerular damage and fibrosis that was completely ameliorated with RAS blockade (Chen et al., 2023), further supporting a deleterious role of impaired TGF during low nephron number and the importance of efferent arteriolar vasodilation even if that therapy further impairs TGF (Chen et al., 2023). Interestingly, CCBs that vasodilate both afferent and efferent arterioles significantly reduce proteinuria in hypertensive CKD patients and have similar renoprotection as RAS blockade (Hayashi et al., 2003).

In the current study, we originally hypothesized that in a virtual AA population with hypertensive CKD, CCB would exacerbate glomerular pressures and increase damage nephrons. Interestingly, opposing this hypothesis, compared to the control models (uncontrolled hypertensive CKD), CCB had similar glomerular pressures and functional nephrons as compared to control albeit at lower systemic BP (Figure 6). These results suggest dihydropyridine CCB man not exacerbate glomerular HTN if BP lowering is achieved. The ability to control BP gets more difficult as CKD progresses, highlighted by the fact the prevalence of resistant HTN is 2–3x more likely in moderate to advanced CKD patients (Thomas et al., 2016; Georgianos and Agarwal, 2020). Whether the virtual population was treated with CCB or dual therapy, high baseline SBP was a significant predictor of glomerular damage (Figure 7). Interestingly, greater decreases in BP with CCB treatment correlated with lower glomerular pressure and nephron damage rate (Figure 7), suggesting intensive BP therapy may be beneficial in at risk CKD patients even when a dihydropyridine CCB is being used. Indeed, the AASK trial demonstrated that more intensive BP therapy significantly lowered proteinuria, a predictive index of glomerular damage and CKD progression, although the clinical composite outcome (GFR decline, ESRD, or mortality) was only significant in the intensive BP group (∼120 mmHg systolic BP) if the patients had evidence of baseline glomerular damage/proteinuria (Appel et al., 2010). When simulating RAS blockade, the amount of BP lowering did not dictate the amelioration of damaged nephrons. These data support the results from the AASK trial in that intensive BP lowering is an important factor in preserving renal function/filtering nephrons but predicts that this may only be an important during CCB therapy or in the absence of RAS inhibition during hypertensive CKD treatment.

There are several unknowns that remain in the treatment of hypertensive CKD. First, CCB still exists as a possible first-line therapy in treating HTN as long as the CKD patient does not show signs of overt proteinuria (in which case RAS blockade is recommended). Most evidence highlighting the renoprotective effects of RAS blockade during CKD is in the diabetic population. In diabetes, ARB was shown to reduce clinical end points of CKD progression by 16% (Tapia et al., 2012). However, the evidence and recommendations for RAS blockade for first-line therapy in nondiabetic CKD patients is not as clear (Kidney Disease: Improving Global Outcomes Glomerular Diseases Work G, 2021). The AASK trial showed a 38% reduction in CKD progression end points with ACE inhibition as compared to CCB (Wright et al., 2002). Additionally, there have been few clinical studies focusing on the renoprotective effect of CCB + RAS blocker combination therapy in non-diabetic CKD. In a mostly diabetic population (60%), the ACCOMPLISH Trial (n = 11,506) demonstrated that there was a slower decline in eGFR with *CCB* and RAS inhibition than *diuretic* and RAS inhibition (Bakris et al., 2010) but this has not been confirmed by others (Bakris et al., 2008; Faucon et al., 2023). The CREOLE trial investigated CCB + RAS inhibition in AA patients (without CKD) and found a robust 18 mmHg fall in ambulatory systolic BP after 6 months (Ojji et al., 2022). They noted eGFR stayed relatively stable at 98 mL/min, but this combination therapy remains largely untested in the AA CKD population.

The efficacy of lowering salt in the AA CKD population is also unclear, especially during antihypertensive therapy. Our model predicted −6 mmHg additional BP lowering from RAS inhibition and another −3 mmHg with lowering salt intake to 90 meq/day (Supplementary Figure S2). Clinically lowering sodium intake plays a significant role in BP control in CKD patients. For example, lowering from 160 to 90 meq/day in Stage 3–4 CKD (∼35 mL/min eGFR) results in ∼10 mmHg fall in ambulatory systolic BP (Saran et al., 2017). Also, low salt diets can add favorable synergistic effects from RAS blockade: low salt diet during CKD results in increased activation of the RAS and a better response to RAS blockers, improvements in both BP and proteinuria control (Slagman et al., 2011). The AASK trial demonstrated a significant association between salt intake and left ventricular mass: 40 mEq/day increase in salt intake translating to a 9 g increase in LV mass, although there was no significant effect on morbidity or mortality (Pearson et al., 2020). Interestingly, LV mass was not a variable used to calibrate the model, but predictions in the decrease in LV mass (3.4% ± 6%) in the virtual population was within range of the results from the CCB arm of the AASK trial (3.7% ± 24%). Reducing salt to 90 meq/day in the virtual population, had additive benefit in both decreasing BP and LV mass (Supplementary Figure S2), confirming the synergistic effects of low salt diet and RAS blockade and represents another important prediction made by the model.

## Limitations

The current virtual population is validated with systemic BP and GFR responses to CCB during CKD. All other predictions with the model should be considered hypotheses until confirmed by experimental studies. The ±5% range that was used to create the virtual population is the largest range we were able to vary all parameters of the model and still consistently retaining model stability. Also, there were no pharmacodynamic or pharmacokinetic data used to calibrate the model and will be the subject of future studies. Glomerular HTN and glomerulosclerosis are associated with proteinuria in experimental models while clinical studies use proteinuria as an index of glomerular damage and/or glomerular HTN. However, the current virtual trial did not include proteinuria. Also, as CKD progresses to more advanced stages and eventually end stage kidney disease, the evidence for RAS blockade on both renoprotection and risks such as hyperkalemia becomes less clear (Weir et al., 2018). Future simulations will investigate these issues by examining the role of both CCB and/or RAS blockade during more advanced CKD stages and dialysis.

## Data Availability

The raw data supporting the conclusion of this article will be made available by the authors, without undue reservation.
