# Supplementary material for: Predicting chronic responses to calcium channel blockade with a virtual population of African Americans with hypertensive chronic kidney disease
Source: Front Syst Biol. 2024 Jul 4;4:1327357. doi: 10.3389/fsysb.2024.1327357 (PMC11600446; doi:10.3389/fsysb.2024.1327357)
Supplement: Supplementary file 1 [file DataSheet1.PDF]

Online Supplement

Predicting chronic responses to calcium channel blockade with a virtual population of African Americans with hypertensive chronic kidney disease

John S. Clemmer, W. Andrew Pruett, and Robert L. Hester

Department of Physiology and Biophysics
University of Mississippi Medical Center
Jackson, MS 39216

Address correspondence to:
John S. Clemmer, PhD
Department of Physiology and Biophysics
University of Mississippi Medical Center
2500 North State Street
Jackson, Mississippi 39216-4505
Email: jclemmer@umc.edu

Key Words: African American, hypertension, kidney disease, physiological model

Supplementary Figure 1. Equations and model parameters for calculating glomerular filtration rate and nephron injury

**Variable**	**Input**
Colloid Osmotic Pressure =	Plasma Osmotic Pressure / (1 - FF)
Bowman's Capsule Pressure =	SNGFR / PTconductance
Capillary Hydrostatic Pressure =	(RBF / Efferent Conductance) + Renal Venous Pressure

$$\mathbf{GFR = Kf\ (P_{C} - P_{BC} - P_{osm})}$$

*FF = GFR / RPF

*SNGFR = GFR / Nephron#

PTconductance = 2.55×10^{-6}

Kf = 8.89 x Nephron# (xNormal)

$$\mathbf{DamageNephron = \int P_{C}}$$

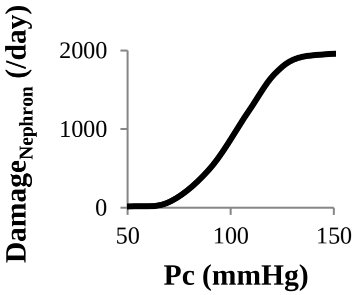

DamageNephron is a function that dictates the decline in functioning/filtering nephrons. FF indicates filtration fraction; SNGFR, single nephron glomerular filtration rate; PTconductance, conductance of the proximal tubule; RBF, renal blood flow; Kf, filtration coefficient; P_C , capillary hydrostatic pressure; P_{BC}, Bowman's Capsule hydrostatic pressure; P_{osm} , capillary colloid osmostic pressure; and RPF, renal plasma flow.
*Indicates implicit equation

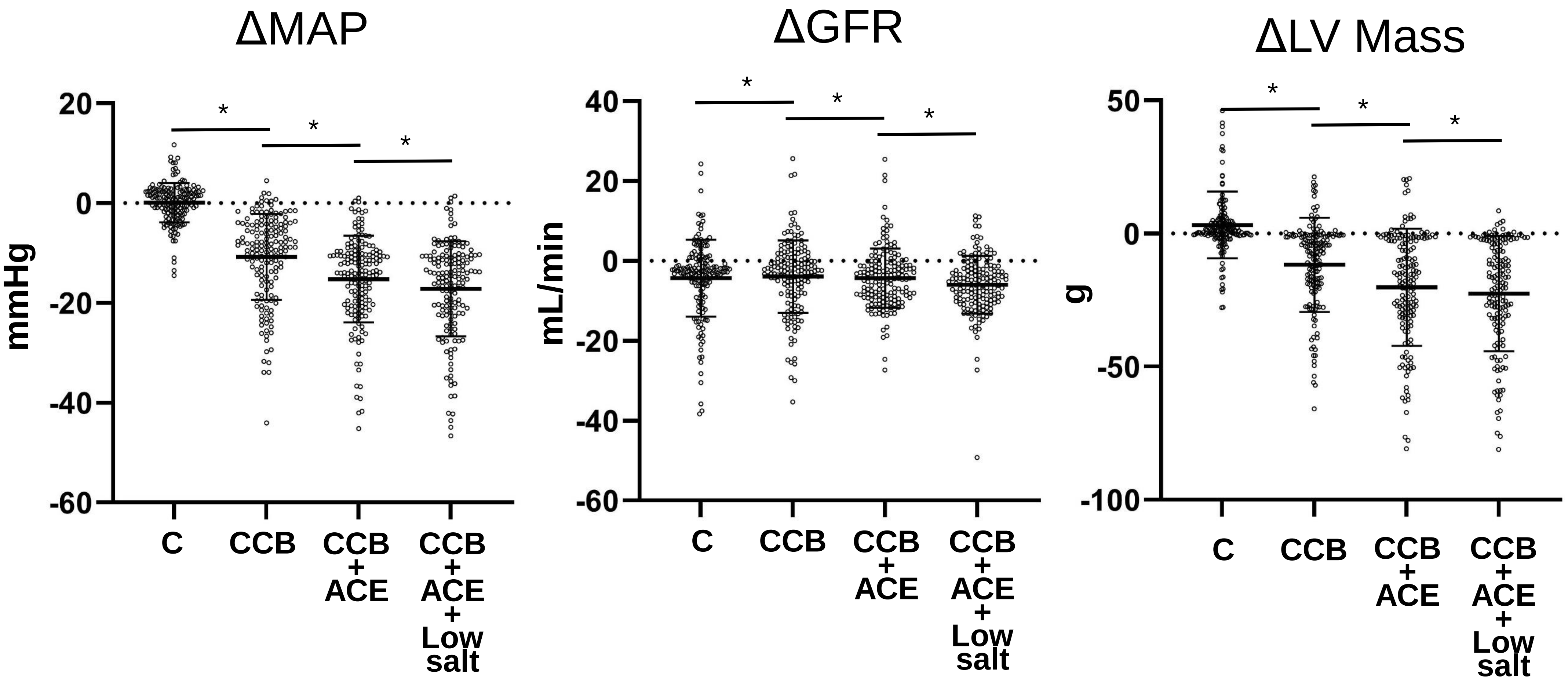

Supplementary Figure S2. Responses to 3 year calcium channel blockade (CCB), ACE inhibition, and/or lowering salt intake (90 mmol Na+/day) in systemic pressure glomerular filtration rate (GFR), and left ventricular mass. C indicates control simulation without any treatment for 3 years. *p<0.05

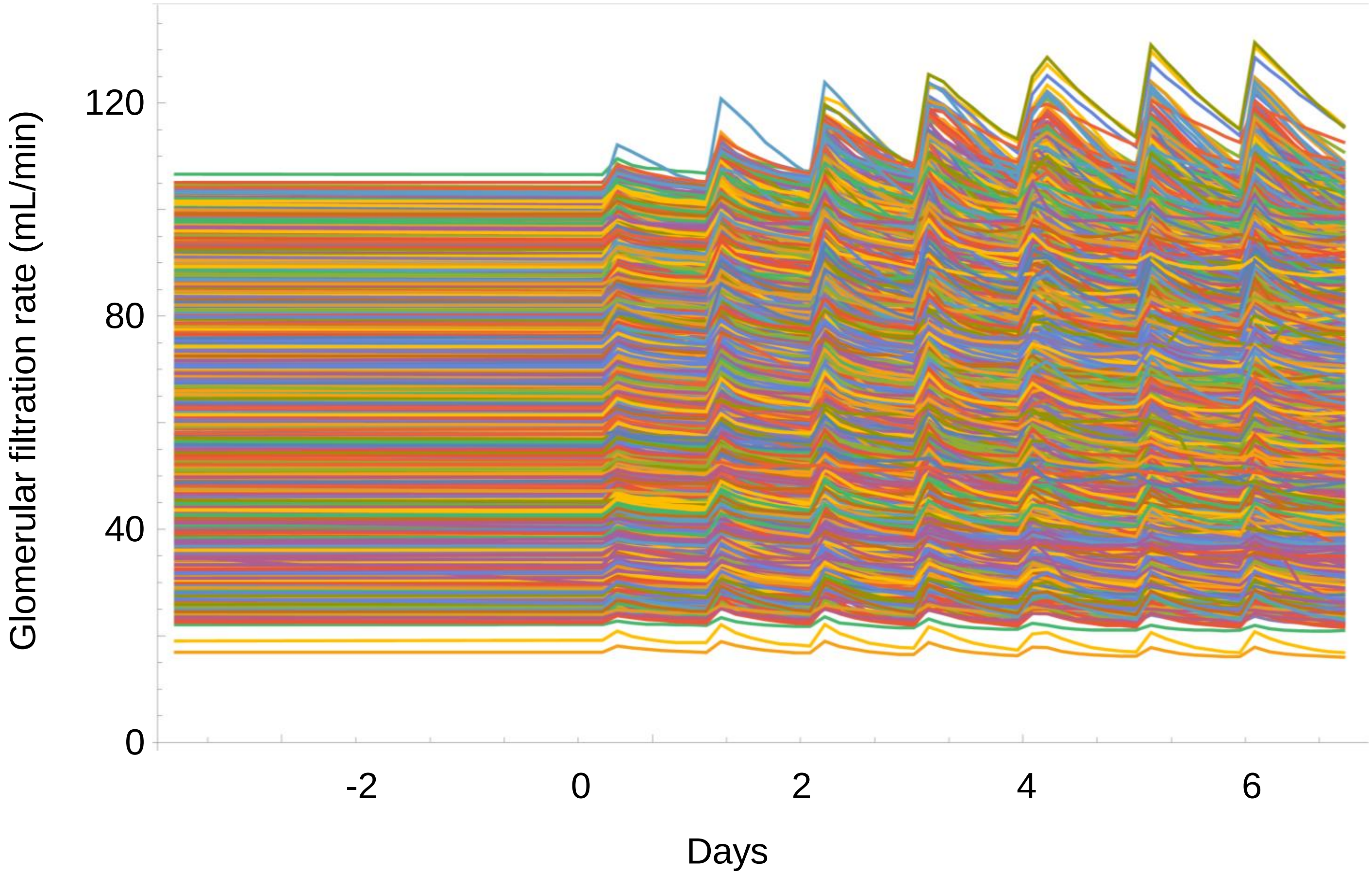

Supplementary Figure S3. Acute changes in glomerular filtration rate after initiation of amlodipine (10 mg/day) in virtual population (n=165)

Supplementary Figure S4. Determinants of renal vasculature: afferent and efferent arteriolar conductance

Afferent arteriolar conductance

$$Conductance = Baseline \times Symp\ Effect \times ANP\ Effect \times Myogenic\ Effect \times TGF\ Effect \times CCB\ Effect$$

$$Baseline = 0.080883 \quad ml/min/mmHg/g$$

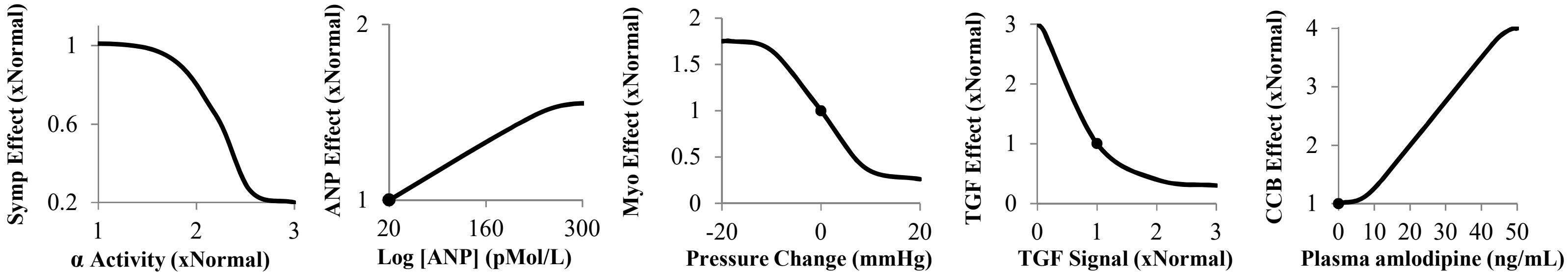

Efferent arteriolar conductance

$$Conductance = Baseline \times Symp\ Effect \times Ang\ II\ Effect$$

$$Baseline = 0.06218 \quad ml/min/mmHg/g$$

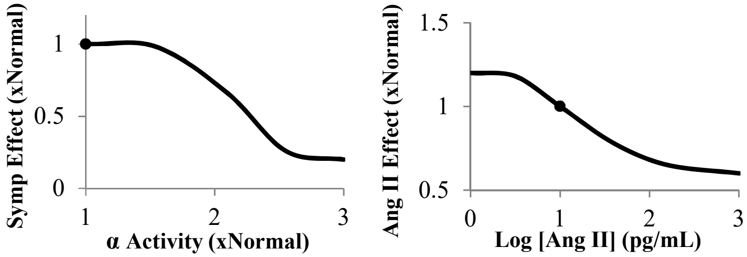

Ang II indicates angiotensin II; ANP, atrial natriuretic peptide; Symp, sympathetic nervous system; myo, myogenic; TGF, tubuloglomerular feedback; and CCB, calcium channel blocker. Baseline values for normal conditions are indicacted in each relationship.

Supplementary Figure S5. Determinants of proximal tubular sodium reabsorption

Proximal tubule sodium reabsorption

$$Reabsorption = PTNa\ Inflow\ \times Baseline^{\ Effects^{-1}}$$

$$Baseline = 58\%$$

$$Effects = \boldsymbol{Ang\ II\ Effect}\ \times \boldsymbol{Symp\ Effect}\ \times \boldsymbol{ANP\ Effect}\ \times \boldsymbol{RIFP\ Effect}$$

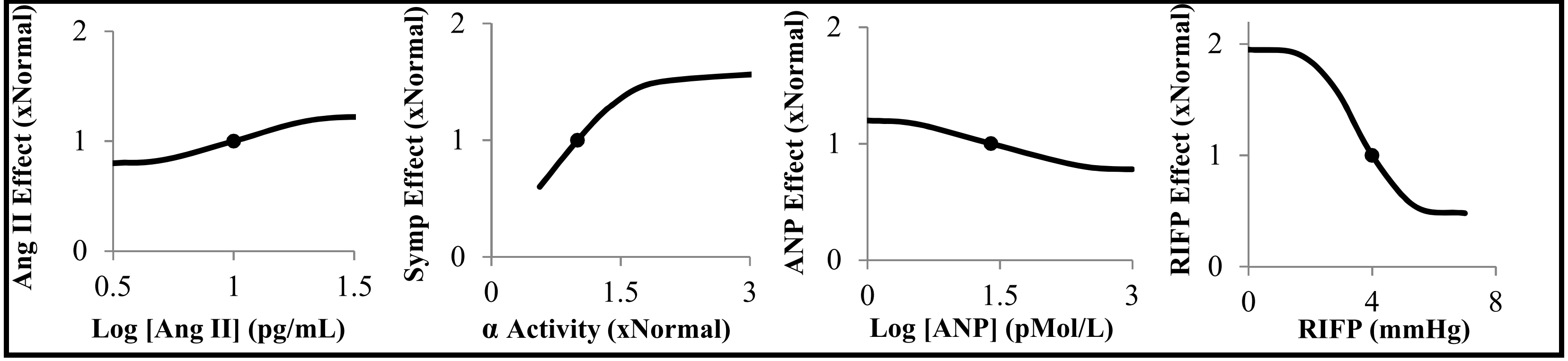

Proximal tubular sodium indicated by PTNa; angiotensin II, Ang II; sympathetic, symp; atrial natriuretic peptide, ANP; renal interstitial fluid pressure, RIFP.

Supplementary Figure S6. Determinants of loop of henle sodium reabsorption

Loop of henle sodium reabsorption

$$Reabsorption = Loop\ Inflow \times Baseline^{\ Effects^{-1}}$$

$$Baseline = 75\%$$

$$Effects = \boldsymbol{Sodium\ Effect} \times \boldsymbol{Flow\ Effect} \times \boldsymbol{Aldo\ Effect}$$

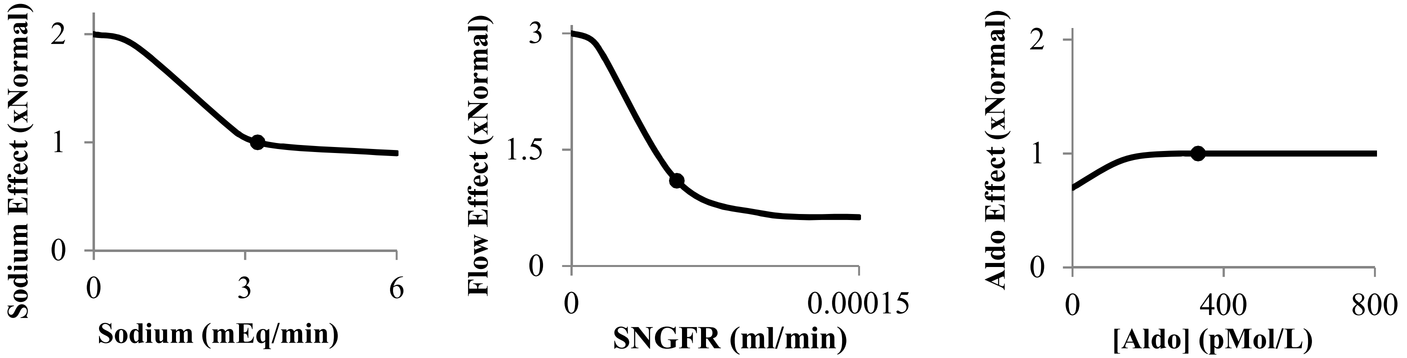

Single nephron glomerular filtration rate, SNGFR; aldosterone, Aldo

Supplementary Figure S7. Determinants of distal tubular sodium reabsorption

Distal tubule sodium reabsorption

$$Reabsorption = Loop\ Inflow \times Baseline^{Effects^{-1}}$$

$$Baseline = 75\%$$

$$Effects = \boldsymbol{Sodium\ Effect} \times \boldsymbol{Aldo\ Effect}$$

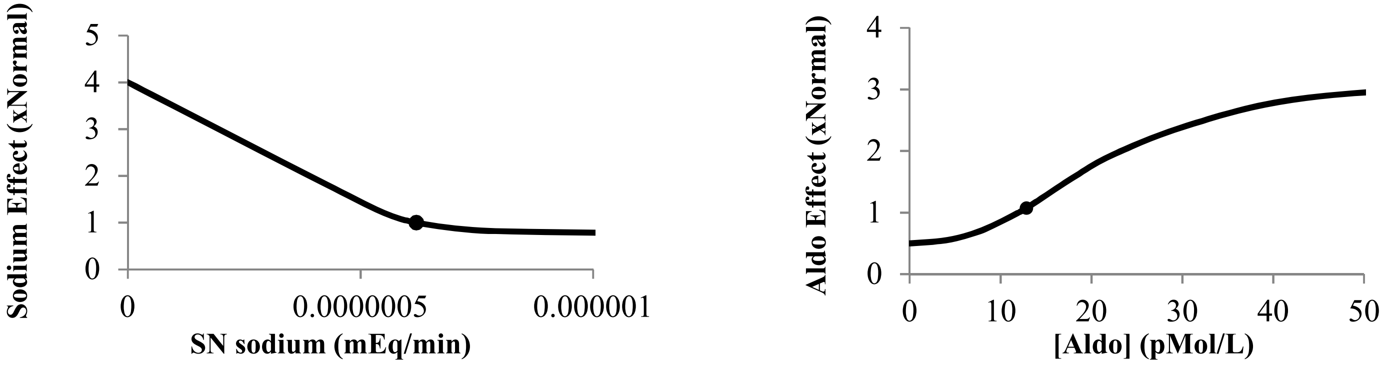

Single nephron, SN; aldosterone, Aldo

Supplementary Figure S8. Determinants of collecting duct sodium reabsorption

Collecting duct sodium reabsorption

$$Reabsorption = Loop\ Inflow \times Baseline^{\ Effects^{-1}}$$

$$Baseline = 75\%$$

$$Effects = \boldsymbol{Sodium\ Effect} \times \boldsymbol{ANP\ Effect}$$

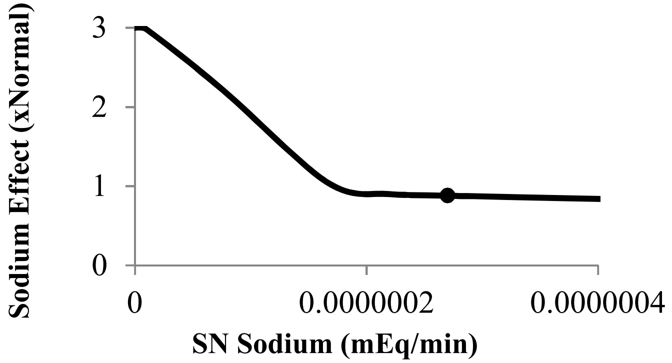

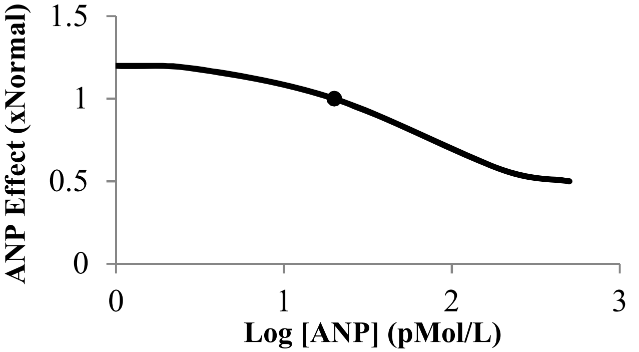

Single nephron, SN; aldosterone, Aldo

Supplementary Figure S9. Determinants of total body water and balance between body fluid compartments

Total Body Water

$$\mathbf{TBW = Tissue_{H2O} + Plasma_{Vol} + RBC_{H2O}}$$

$$\mathbf{Tissue_{H2O} = Torso_{Upper} + Torso_{Middle} + Torso_{Lower}}$$

$$\mathbf{\frac{dTorso_{Upper,\ Middle,\ Lower}}{dt} = Intracellular + Interstitial = CapillaryFiltration + Metabolism_{H2O} - Lymph_{Rate} - Loss_{H2O}}$$

$$\mathbf{Plasma_{Vol} = GI\ Absorption + Lymph_{Rate} - Urine - CapillaryFiltration}$$

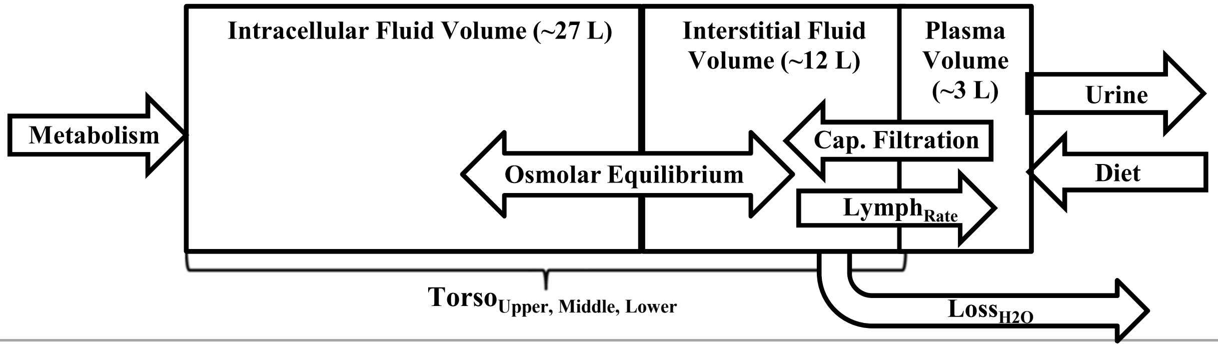

Tissue H_2O calculated for each torso region:

$$\mathbf{Gain_{H2O} = CapillaryFiltration + Metabolism}$$

$$\mathbf{Loss_{H2O} = Sweat + Skin_{ins} + Lymph_{Rate}}\ \textit{(+ Lung for Torso}_{Middle}\textit{)}$$

$$\mathbf{CapillaryFiltration = Perm \times [P_{capillary} + COP_{interstitial} - P_{interstitial} - COP_{capillary}]}$$

$$\mathbf{Lymph_{Rate} = K \times [P_{interstitial} \times Effect]}$$

$Perm_{Upper}$ = 1.16075 ml/mmHg/min

$Perm_{Middle}$ = 2.5374 ml/mmHg/min

$Perm_{Lower}$ = 1.7006 ml/mmHg/min

K_{Upper} = 4.43

K_{Middle} = 8.73

K_{Lower} = 4.6

Supplementary Figure S10. Determinants of renin secretion and macula densa signal

Renin Secretion

$$Renin\ Synthesis = \boldsymbol{MD\ Effect} \times \boldsymbol{Symp\ Effect} \times Renal\ Mass\ \% \times Base$$

$$Renin\ Mass = Renin\ Synthesis - Renin\ Secretion$$

$$Renin\ Secretion = \boldsymbol{MD\ Effect} \times \boldsymbol{Symp\ Effect} \times Renin\ Mass \times K$$

$$Base\ synthesis = 160\ GU/min$$

$$K = 0.00165$$

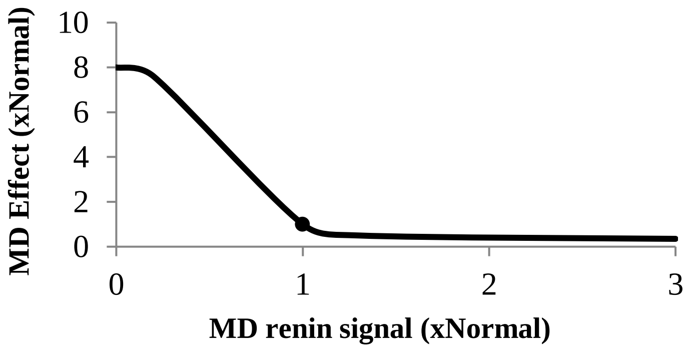

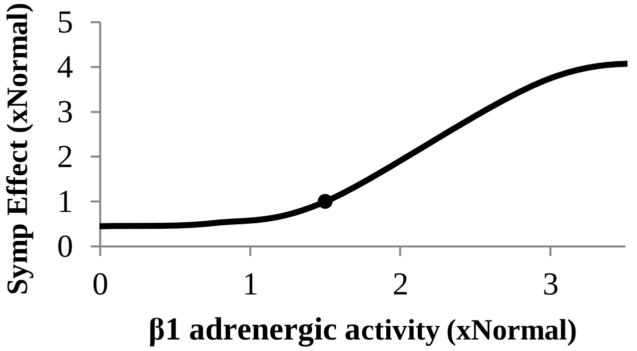

Macula densa renin signal

$$Signal = \boldsymbol{Sodium\ Effect} \times \boldsymbol{Ang\ II\ Effect} \times \boldsymbol{ANP\ Effect}$$

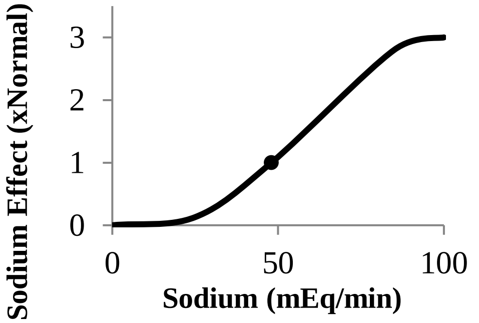

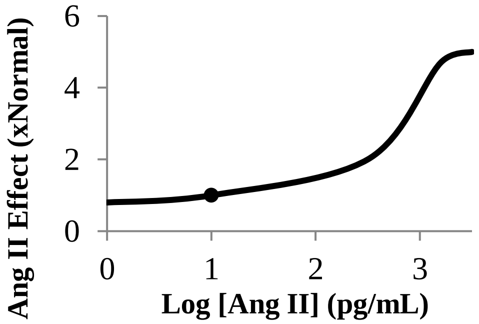

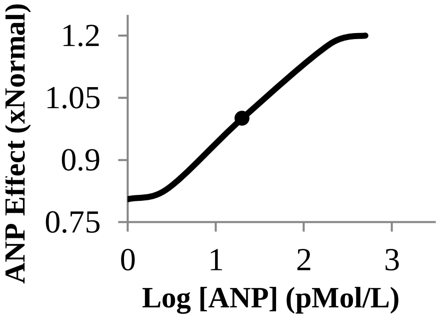

Symp indicates sympathetic; MD, macula densa; Ang II, angiotensin II; ANP, atrial natriuretic peptide. Baseline value for normal model conditions are indicated in each relationship.

Supplementary Figure S11. Determinants of tubuloglomerular feedback

TGF signal

$$Signal = \boldsymbol{Sodium\ Effect} \times \boldsymbol{Ang\ II\ Effect} \times \boldsymbol{ANP\ Effect}$$

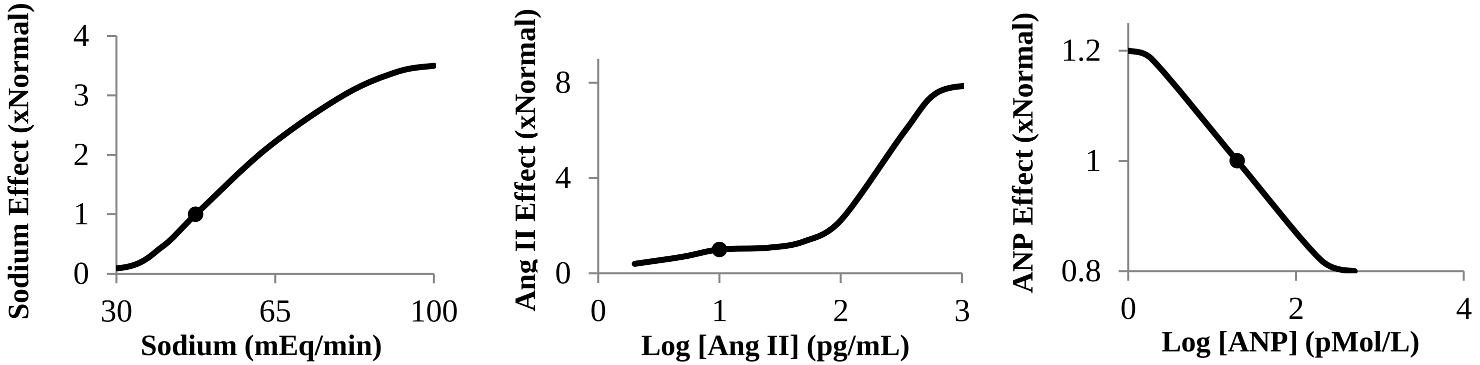

TGF indicates tubular glomerular feedback Angiotensin II, Ang II; atrial natriuretic peptide, ANP

Supplementary Figure 12. Peripheral blood flow and determinants of organ conductances in the model

Skeletal Muscle Conductance		Effect Range	Input
	**Ang II Effect***	0.5 - 1.05	[Ang II]
	**Sympathetic Effect***	0.3 - 1.3	SM α_1 Receptor Activation
	**Sympathetic Effect**	0.5 - 1.75	SM β_2 Receptor Activation
	**Delayed PO_2 Effect***	0 - 1.2	SM PO_2
	**Acute PO_2 Effect***	0.4 - 6	SM PO_2
	**ADH Effect***	0.1 - 1	[ADH]
	**Metabolism Effect**	1.0 - 3	Metabolic Oxygen Need
	**Muscle Pump Effect**	1.0 - 3	Intensity and Rate of Exercise
	**CCB Effect**	1.0 - 4	[Amlodipine]
Baseline conductance (ml/min/mmHg/g) = 0.00029			

GI Tract Conductance		Effect Range	Input
	**Ang II Effect***	0.5 - 1.05	[Ang II]
	**Sympathetic Effect***	0.1 - 1.3	GI α_1 Receptor Activation
	**Delayed PO_2 Effect***	0.2 - 1.2	GI PO_2
	**Acute PO_2 Effect***	0.2 - 2	GI PO_2
	**ADH Effect**	0.1 - 1	[ADH]
	**CCB Effect**	1.0 - 4	[Amlodipine]
Baseline conductance (ml/min/mmHg/g) = 0.00904			

Fat Conductance		Effect Range	Input
	**Ang II Effect***	0.5 - 1.05	[Ang II]
	**Sympathetic Effect***	0.1 - 1.3	Fat α_1 Receptor Activation
	**ADH Effect***	0.1 - 1	[ADH]
	**Delayed PO_2 Effect***	0.8 - 1.2	Fat PO_2
	**Acute PO_2 Effect***	0.4 - 2	Fat PO_2
	**CCB Effect**	1.0 - 4	[Amlodipine]
Baseline conductance (ml/min/mmHg/g) = 0.00019			

Bone Conductance		Effect Range	Input
	**Ang II Effect***	0.5 - 1.05	[Ang II]
	**Sympathetic Effect***	0.1 - 1.3	Bone α_1 Receptor Activation
	**ADH Effect***	0.1 - 1	[ADH]
	**Delayed PO_2 Effect***	0.8 - 1.2	Bone PO_2
	**Acute PO_2 Effect***	0.4 - 2	Bone PO_2
Baseline conductance (ml/min/mmHg/g) = 0.00029			

Ang II indicates angiotensin II; SM, skeletal muscle; PO_2, partial pressure of oxygen; ADH, antidiuretic hormone; GI, gastrointestinal; PCO_2 , partial pressure of carbon dioxide; and temp, temperature.

*Indicates a negative relationship

Supplementary Figure 12b (continued). Peripheral blood flow and determinants of organ conductances

Brain Conductance		Effect Range	Input
	**Delayed PO_2 Effect***	0.8 - 1.2	Brain PO_2
	**Acute PO_2 Effect***	0.9 - 2.2	Brain PO_2
	**Acute PCO_2 Effect**	0.7- 2.2	Brain PCO_2

Baseline conductance (ml/min/mmHg/g) = 0.00597

Skin Conductance		Effect Range	Input
	**Ang II Effect***	0.5 - 1.05	[Ang II]
	**Sympathetic Effect***	0.1 - 1.3	Other Tissue α_1 Receptor Activation
	**Body Temp Effect**	0.3 - 8	(Core Temp - 37 °C)
	**Local Temp Effect**	0.2 - 5	Skin Temp
	**ADH Effect***	0.1 - 1	[ADH]
	**Delayed PO_2 Effect***	0.8 - 1.2	Skin PO_2
	**Acute PO_2 Effect***	0.2 - 2	Skin PO_2

Baseline conductance (ml/min/mmHg/g) = 0.00112

Liver Conductance		Effect Range	Input
	**Sympathetic Effect***	0.1 - 1.3	Hepatic Artery α_1 Receptor Activation

Baseline conductance (ml/min/mmHg/g) = 0.00187

Other Tissue Conductance		Effect Range	Input
	**Ang II Effect***	0.5 - 1.05	[Ang II]
	**Sympathetic Effect***	0.1 - 1.3	Tissue α_1 Receptor Activation
	**ADH Effect***	0.1 - 1	[ADH]
	**Delayed PO_2 Effect***	0.8 - 1.2	Tissue PO_2
	**Acute PO_2 Effect***	0.2 - 2	Tissue PO_2
	**CCB Effect**	1.0 - 4	[Amlodipine]

Baseline conductance (ml/min/mmHg/g) = 0.00141

Ang II indicates angiotensin II; SM, skeletal muscle; PO_2, partial pressure of oxygen; ADH, antidiuretic hormone; GI, gastrointestinal; PCO_2, partial pressure of carbon dioxide; and temp, temperature.

*Indicates a negative relationship
